# Partitioning Carbon Dioxide Emission and Assessing Dissolved Organic Carbon Leaching of a Drained Peatland Cultivated with Pineapple at Saratok, Malaysia

**DOI:** 10.1155/2014/906021

**Published:** 2014-08-19

**Authors:** Liza Nuriati Lim Kim Choo, Osumanu Haruna Ahmed

**Affiliations:** ^1^Department of Crop Science, Faculty of Agriculture and Food Science, Universiti Putra Malaysia (UPM), Bintulu Campus, P.O. Box 396,97008 Bintulu, Sarawak, Malaysia; ^2^Soil and Water Management Programme, Strategic Resource Research Centre, Malaysian Agricultural Research and Development Institute (MARDI), P.O. Box 59, Roban, 95300 Saratok, Sarawak, Malaysia

## Abstract

Pineapples (*Ananas comosus* (L.) Merr.) cultivation on drained peats could affect the release of carbon dioxide (CO_2_) into the atmosphere and also the leaching of dissolved organic carbon (DOC). Carbon dioxide emission needs to be partitioned before deciding on whether cultivated peat is net sink or net source of carbon. Partitioning of CO_2 _ emission into root respiration, microbial respiration, and oxidative peat decomposition was achieved using a lysimeter experiment with three treatments: peat soil cultivated with pineapple, bare peat soil, and bare peat soil fumigated with chloroform. Drainage water leached from cultivated peat and bare peat soil was also analyzed for DOC. On a yearly basis, CO_2_ emissions were higher under bare peat (218.8 t CO_2_ ha/yr) than under bare peat treated with chloroform (205 t CO_2_ ha/yr), and they were the lowest (179.6 t CO_2_ ha/yr) under cultivated peat. Decreasing CO_2_ emissions under pineapple were attributed to the positive effects of photosynthesis and soil autotrophic activities. An average 235.7 mg/L loss of DOC under bare peat suggests rapid decline of peat organic carbon through heterotrophic respiration and peat decomposition. Soil CO_2_ emission depended on moderate temperature fluctuations, but it was not affected by soil moisture.

## 1. Introduction

Tropical peat soils are generally defined as soils formed by the accumulation of partially decayed woody plant materials under waterlogged condition. Tropical peatlands cover 27.1 million hectares in Southeast Asia [1] and about 2.6 million hectares in Malaysia [2]. Peats of the tropics are increasingly being cultivated. Although they store large amount of organic carbon, peat soils drained for agriculture in particular accelerate their decomposition rates. Rapid decomposition of peats leads to increase in CO_2_ release into the atmosphere [3, 4]. Carbon dioxide may be emitted from peatland through burning by wildfires, microbial respiration, root respiration, and physical oxidation [5, 6]. Carbon dioxide emissions are related to water table depth [7], soil temperature [8, 9], fertilization [10], land use type [11], and peat type [12]. Moreover, carbon in the form of DOC is lost through leaching due to microbial metabolism [13]. Carbon losses through emission and leaching may shift the peatland carbon balance from sink to source [14].

In Malaysia, approximately 600,000 hectares of peatland are cultivated with oil palm, pineapple, rubber, and sago [15]. Presently, there is scarce information on soil CO_2_ emission from pineapple cultivation on drained peat soils. The understanding of the contribution of pineapple cultivation on peats to the greenhouse gas emission is important, as 90% of pineapples are grown on peat soils of Malaysia [16]. Although attempts have been made to measure CO_2_ emission from cultivated tropical peats, such studies are limited to a few measurements. The recent measurements only account for total soil CO_2_ emission as they do not partition soil respiration into root respiration, microbial respiration, and oxidative peat decomposition [10, 17]. With the growing concern about the effects of greenhouse gases on the environmental quality coupled with the need to achieve sustainable agriculture, it is essential to partition CO_2_ emission before deciding on whether cultivated or degraded soils are net sinks or net sources of atmospheric greenhouse gases [5]. Accounting for CO_2_ emission from cultivated peats is needed to evaluate future rates of increase in atmospheric greenhouse gases and their effect on the global environmental change processes [18, 19].

Based on the above rationale, the general objective of this study was to quantify CO_2_ emission and also carbon loss from a drained tropical peat grown with pineapple. The first specific objective was to partition soil CO_2_ emission from a cultivated peat into root respiration, microbial respiration, and oxidative peat decomposition. The second specific objective was to estimate DOC in water drained from lysimeters with peat soil. The third specific objective was to assess the effects of soil temperature and soil moisture on soil CO_2_ emission.

In this study, it was hypothesized that microbial respiration and peat decomposition will cause higher loss of CO_2_ and DOC from the bare peat soil than from the peat soil cultivated with pineapple. This hypothesis is based on the assumption that CO_2_ emission of drained and uncultivated peats is mainly controlled by heterotrophic respiration. However, CO_2_ and DOC release in the presence of root respiration (cultivated peats) is expected to be lower as both processes are regulated by autotrophic respiration and photosynthesis. Information obtained from partitioning respiration components could be used to control CO_2_ and DOC losses from drained tropical peats that are cultivated with pineapples and other related crops.

## 2. Materials and Methods

### 2.1. Site Description

The study was carried out at the Malaysian Agricultural Research and Development Institute (MARDI) Peat Research Station at Saratok, Sarawak, Malaysia. The research station has a total area of 387 hectares located on a logged-over forest with a flat topography of 5 to 6 m above mean sea level. Based on the Von Post Scale of H7 to H9, the peat soil is classified as well decomposed dark brown to almost dark coloured sapric peat with a strong smell. The thickness of the peat soil ranges from 0.5 to 3.0 m.

The mean temperature of the peat area ranges from 22.1 to 31.7°C. The relative humidity of the area ranges from 61 to 98%. The annual mean rainfall of the area is 3749 mm. In the wet season (November to January), the monthly rainfall is more than 400 mm whereas in the dry season, particularly in July, the mean rainfall is 189 mm.

### 2.2. Soil Chemical and Physical Analysis

Before setting up the lysimeter experiment, peat samples were collected at a peat excavation site (0.5 hectares) located at MARDI Peat Research Station. The experimental area was planted with* Moris* pineapple from 2004 to 2005, after which it was abandoned to lie fallow for six years. Samplings were performed at depths of 0–20 cm, 20–40 cm, and 40–, 60 cm systematically in 12 points located over a 20 m × 12.5 m grid. The soil samples were analyzed for pH, conductivity, ammonium-N, nitrate-N, organic carbon, total nitrogen, and cation exchange capacity (CEC). Soil pH and conductivity were measured based on a 1 : 5 soil to water suspension [20]. Ammonium-N and nitrate-N were determined using the steam distillation method [21]. Soil organic carbon was determined using the Walkley and Black method [22] whereas total nitrogen was determined using the Kjeldahl method [23]. Cation exchange capacity was determined using the Harada and Inoko method [24]. Bulk density was determined using the core method [25], and soil water holding capacity was determined using the method of Dugan et al. [26].

### 2.3. Characteristics of the Lysimeters

Twelve cylindrical field lysimeters made from high density polyethylene, measuring 1.43 m in diameter and 1.58 m in height, were set up in April 2012 to mimic the natural condition of drained tropical peats. The size of the lysimeters used in this study was designed to ensure satisfactory growth and development of the pineapples for sixteen months. The twelve lysimeters were used for three peat soil treatments (Section 2.4). The lysimeters were equipped with water spillage opening which was attached to clear tubes mounted on the outside of the vessel to regulate and monitor water level.

Each lysimeter was filled with peat soil up to 120 cm depth. Water loss from the soil was replenished by showering each lysimeter with 34.5 litres of rainwater. The amount of rainwater added was based on the volume of the fabricated lysimeter and the mean annual rainfall at Saratok, Sarawak, Malaysia. The lysimeters with the peat soil were left in the open for five months to ensure that the peat soil had settled before beginning this study. The length of this initial phase was based on weekly determination of the peat subsidence. The equilibrium state was achieved in September 2012 before carrying out the CO_2_ measurement. Water table of the peat was maintained at 50 to 60 cm from the soil surface throughout the duration of the experiment.

### 2.4. Peat Soil Treatments

The three treatments involved in this lysimeter experiment were peat soil cultivated with pineapple (A), bare peat soil (B), and bare peat soil treated with chloroform (C). Each treatment had four replications. The treatments were arranged in completely randomized design.

Treatment A represents total amount of CO_2_ emitted from root respiration, microbial respiration, and peat decomposition. Three* Moris* pineapple suckers were planted in the lysimeters at a distance of 30 cm. Treatment B represents CO_2_ emitted by microbial respiration and peat decomposition. Weed sprouting on the soil surface was controlled when necessary. Treatment C represents CO_2_ emitted by oxidative peat decomposition. For this treatment, concentrated chloroform was applied evenly on the peat soil surface to eliminate microbial respiration, and 64.6 litres of concentrated chloroform was used. This volume was based on the peat soil's water holding capacity. After the chloroform application, the soil was covered with cling film and canvas to produce a vacuum-like condition in the lysimeters to minimize chloroform volatilization. The soil microbial population before and after the chloroform application was determined using the culture method. With this method, bacteria, fungi, and actinomycetes were enumerated as colony forming units (CFU) per gram of fresh soil on nutrient agar, Rose Bengal, and actinomycetes isolation agar, respectively [27]. The concentrated chloroform was used to fumigate the peat soil one week before the soil CO_2_ measurement was commenced (optimum time interval achieved for the biocidal effect on soil microorganisms).

### 2.5. Soil CO_2_ Emission Measurements

Carbon dioxide emissions from the field lysimeters were measured using the closed chamber method [28]. Extracted gas samples from the chamber were analyzed for CO_2_ using gas chromatography (Agilent 7890A) equipped with thermal conductivity detector (TCD). The CO_2_ results were based on the measured CO_2_ from treatments A, B, and C in the wet and dry seasons. The values were averaged and converted into units of t/ha/yr. The gas flux was calculated from the increase in the chamber concentration over time using the chamber volume and soil area covered, using the following equation [28–30]:

(1) $$\begin{array}{c} \text{Flux}=\left[\frac{d\left({\text{CO}}_{2}\right)}{dt}\right]\times \frac{PV}{ART}, \end{array}$$

where *d*(CO_2_)/(*dt*) is the evolution rate of CO_2_ within the chamber headspace at a given time after putting the chamber into the soil, *P* is the atmospheric pressure, *V* is the volume headspace gas within the chamber, *A* is the area of soil enclosed by the chamber, *R* is the gas constant, and *T* is the air temperature.

The gas flux was measured in the early morning (2.40 a.m. to 5.55 a.m.), morning (7.15 a.m. to 10.30 a.m.), mid-morning to afternoon (10.35 a.m. to 1.50 p.m.), afternoon (1.55 p.m. to 5.10 p.m.), evening (8.00 p.m. to 11.15 p.m.), and night (11.20 p.m. to 2.35 a.m.) to obtain a 24 hour CO_2_ emission. The flux measurements were carried out in September 2012, November 2012, and January 2013 to represent the concentrations of CO_2_ in the wet season whereas April 2013 and July 2013 flux measurements represent the concentrations of CO_2_ in the dry season. Soil temperature and moisture were measured using Eijkelkamp IP68 and ML3 sensors, respectively. Rainfall, temperature, and air humidity data were also recorded using a portable weather station (WatchDog 2900) installed at the experimental site.

### 2.6. Measurement of DOC

Water draining through the openings of the lysimeters of treatments A and B was collected for determinations of DOC concentration. The water samples were chilled at 10°C before being analyzed. The samples were filtered to pass a 0.45 *μ*m cellulose nitrate membrane filter and the contents of DOC were determined using total carbon analyzer (Shimadzu TOC). The DOC from treatment C was not measured because of the high residual chloroform in the peat water.

### 2.7. Statistical Analysis

Treatment effects were tested using analysis of variance (ANOVA) and means of treatments were compared using Duncan's New Multiple Range Test at *P* ≤ 0.05. The relationships between gas flux, soil temperature, and soil moisture were analyzed using Pearson correlation analysis. The statistical software used for this analysis was the Statistical Analysis System (SAS) Version 9.3.

## 3. Results and Discussion

### 3.1. Peat Physical Properties

Results of peat soil properties are compared with the previously reported ranges (Table 1) for tropical peats in Southeast Asia [31] and Malaysia [31–33]. The bulk density of the sampled peat soil is within the reported range [31] whereas the water holding capacity and moisture content of the soil are lower than the reported range [31, 32]. The bulk density at 10 cm ranged from 0.09 to 0.18 g/cm^3^, which is typical of a sapric peat. The bulk density was determined at 10 cm due to the saturated condition of the excavation site. The water holding capacity of the peat (40.2%) was below the reported range [31] because the water holding capacity determination was based on oven-dry weight method [31]. The increasing moisture content with increasing peat soil depth is related to the high water table at the excavation site during soil sampling. However, the soil moisture is lower than the reported range [32]. Removal of trees and debris after land clearing may have accelerated oxidative peat decomposition and therefore soil moisture content is lower.

### 3.2. Peat Chemical Properties

Values of pH, conductivity, CEC, total organic carbon, and total nitrogen of the peat soil studied here are within the reported range [31–34] for the peat soil at MARDI Peat Research Station (Table 1). The soil chemical properties showed no significant difference with depth except for total nitrogen, ammonium-N, and nitrate-N. The pH of the peat soil was low, suggesting a need for liming before being cultivated. The low conductivity of the peat soil also indicates that the soil is not saline as the research station is drained by two large tidal rivers (Sebelak River and Nyabor River). However, intrusion of salt water at the station is prevented by a tidal gate constructed at the main outlet drain leading to Nyabor River. The CEC of the peat soil is high because of lignin-derivatives formed during decomposition. Ion exchange in peats relates to carboxyl and phenolic radicals of humic substances and hemicelluloses [31]. However, the CEC obtained is higher than the reported range [33]. This may be attributed to the past liming activities at the excavation site as this area was cultivated with pineapples from 2004 to 2005. The total organic carbon of the soil is within the reported range [31, 34]. The high organic carbon content can be associated with the botanical origin (woody) of the sapric peat used in this study [31, 32]. The total nitrogen of the soil was high and it was mostly in organic form. The total nitrogen ranged from 1.1 to 1.3%. Ammonium-N ranged from 94.8 to 138.5 mg/L whereas nitrate-N ranged from 48.8 to 72.0 mg/L at the three soil depths. Total nitrogen, ammonium-N, and nitrate-N contents decreased with increasing soil depth (from 0–20 cm to 20–40 cm depths) because decomposition of peats generally decreases (low oxidation with increasing water content) down the soil profile [31].

### 3.3. Soil CO_2_ Emission

**Figure 1:**
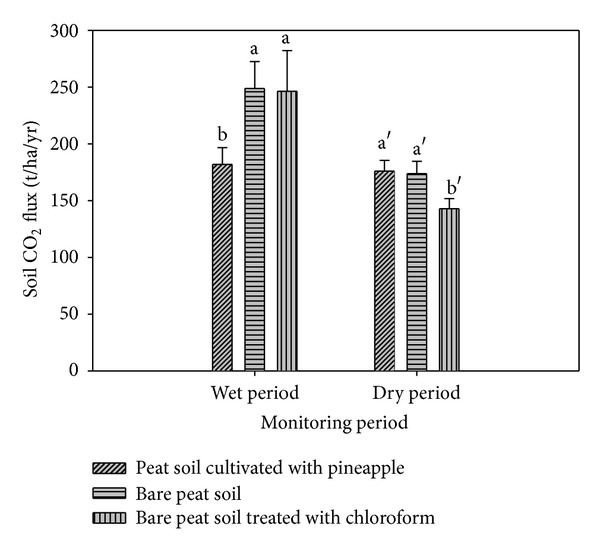
Carbon dioxide emission (wet and dry seasons) from peat soil cultivated with pineapple, bare peat soil, and chloroform fumigated peat soil. (Error bars represent standard error and soil mean fluxes with different letters are significantly different at *P* ≤ 0.05.)

The CO_2_ emissions under treatments A, B, and C varied in the wet and dry seasons (Figure 1). In the wet season, the CO_2_ emission under treatment A was significantly lower than under treatments B and C. However, in the dry season, the CO_2_ emission under treatment C was significantly lower than under treatments A and B. The CO_2_ emission under treatment A was affected by root development and growth of the pineapple plants. Differences in day and night temperatures in the wet and dry seasons (Table 2) may have also impeded photosynthetic activity of the pineapple plants [35]. Furthermore, heterotrophic respiration and decomposition of root exudates in the rhizosphere [5, 36] may have contributed to the CO_2_ emission under treatment A.

The CO_2_ emission under treatment B is related to the microbial population of the peat soil and the availability of adequate substrate for microbial metabolism, but not to plant root activities [5, 12]. The CO_2_ emission under treatment B was also regulated by moderate temperature fluctuation (Table 2), which is in agreement with previous studies in peat soils [9, 12]. The effect of soil temperature on CO_2_ emission from peat soils has been also recently studied by Jauhiainen et al. [8] and Paz-Ferreiro et al. [37], showing that the rate of organic material decomposition increased with increasing temperature of peat soils.

The CO_2_ emission under treatment C was mainly due to oxidative peat decomposition (shrinkage and consolidation) as the fumigant (chloroform) used inhibited microbial respiration. Bacteria and actinomycetes populations before and after fumigation were statistically similar. Fungi were not detected in this present study. These findings are in agreement with most previous findings, which demonstrate that chloroform can effectively kill (94% to 99%) microorganisms [38–42]. The effectiveness of the fumigation is supported by the decrease in the mean soil microbial biomass carbon (Table 3). This result also corroborates previous work by Zelles et al. [43] who reported 80% reduction in microbial biomass carbon after fumigating a soil with chloroform. The fact that the subsidence rates of the peat soil in treatments A, B, and C were statistically similar suggests that the chloroform used did not affect CO_2_ emission due to oxidative peat decomposition. This observation corroborates that of Toyota et al. [40] who also found no significant effect of chloroform fumigation on soil bulk density and compaction. The higher CO_2_ emission under treatment C in the wet season was due to the decomposition of dead microorganisms [42]. In contrast, the decrease in the CO_2_ emission under treatment C in the dry season was because of the adaptation of the microorganisms towards the biocidal effect of chloroform. Again, it must be stressed that the CO_2_ emission was mainly because of oxidative peat decomposition [1, 6]. The oxidative peat decomposition (shrinkage and consolidation) was due to the loss of water in the aerobic layer of the peat. Oxidative peat decomposition is a continuous process and it takes several years to achieve the equilibrium state of peat subsidence.

**Figure 2:**
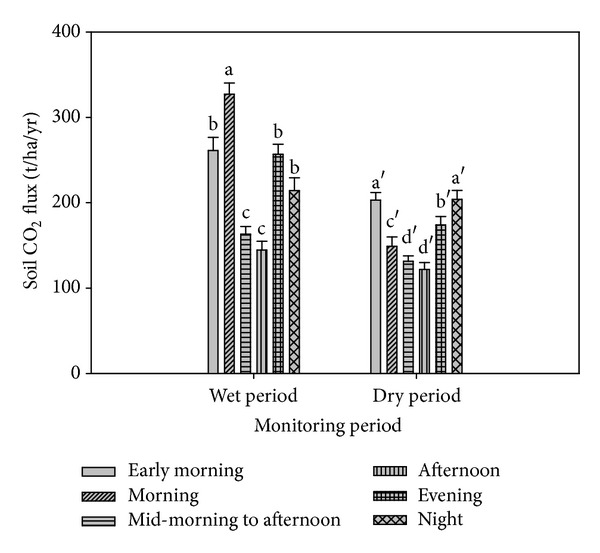
Carbon dioxide emission (at different times of the day and different seasons) from peat soil cultivated with pineapple. (Error bars represent standard error and soil mean fluxes with different letters are significantly different at *P* ≤ 0.05.)

The CO_2_ emission was also affected by time of sampling (Figure 2). In the wet season, the CO_2_ emission decreased from morning to mid-morning to afternoon followed by an increase in the evening. In the dry season, the CO_2_ emission decreased from the early morning to mid-morning to afternoon followed by an increase in the evening and night. These observations are consistent with the significant negative correlation between soil CO_2_ emission and soil temperature (Table 4). These findings also suggest that the CO_2_ emission increased with decreasing temperature. Although the CO_2_ emission was negatively correlated with soil temperature, the overall data (wet and dry seasons) showed no correlation between CO_2_ emission and soil temperature (Table 4). This indicates that although soil temperature regulates soil CO_2_ emission, the differences in the CO_2_ emissions under treatments A, B, and C across time rather depends on the moderate fluctuation in soil temperature (0.2 and 1.6°C) of the tropics. There was no correlation between CO_2_ emission and soil moisture (Table 4) because the water table in the lysimeters was maintained at 50 and 60 cm. This finding is further supported by the fact that the soil moisture was not significantly affected by time of sampling (Table 5). In a related study, Kechavarzi et al. [12] found that soil moisture had no effect on CO_2_ emission in humified peat surface but higher soil moisture which constrained oxygen diffusion affected soil respiration.

In summary, the CO_2_ emission was estimated at about 218.8 t CO_2_ ha/yr under bare peat soil (B), followed by 205 t CO_2_ ha/yr under bare peat soil treated with chloroform (C), and 179.6 t CO_2_ ha/yr under peat soil cultivated with pineapple (A). The higher CO_2_ emission from treatment B suggests that it is controlled by heterotrophic respiration whereas the lower CO_2_ emission from treatment A suggests that it is regulated by autotrophic respiration (through photosynthetic activity and respiration in the rhizosphere). The CO_2_ emission in this study was higher than that reported by Jauhiainen et al. [8], who found that microbial respiration contributed with 80 t CO_2_ ha/yr whereas root respiration contributed with 21% of the total respiration. The CO_2_ emission rate reported in this study is not consistent with that of Jauhiainen et al. [8] because the present study was carried out on sapric peat whereas that reported by Jauhiainen et al. [8] was on fibric to hemic peat.

### 3.4. Dissolved Organic Carbon

**Figure 3:**
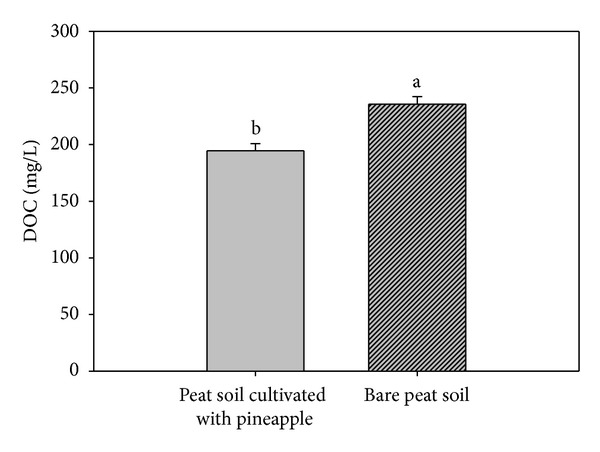
Dissolved organic carbon (DOC) leaching losses under peat soil cultivated with pineapple and bare peat soil. (Error bars represent standard error, and mean DOC with different letters are significantly different at *P* ≤ 0.05.)

The DOC under the bare peat soil (B) (235.7 mg/L) was significantly higher than under peat soil cultivated with pineapple (A) (194.6 mg/L) (Figure 3) because of the greater decomposition of organic substrate by heterotrophs. One of the byproducts of heterotrophs is DOC [13]. The lower DOC under treatment A is related to the consumption of carbon, nitrogen, and root exudates by microbes at the rhizosphere [5, 44]. The DOC under treatment B is in accordance with the results of our field monitoring as the mean CO_2_ under treatment B (218.8 t CO_2_ ha/yr) was higher than under treatment A (179.6 t CO_2_ ha/yr).

**Figure 4:**
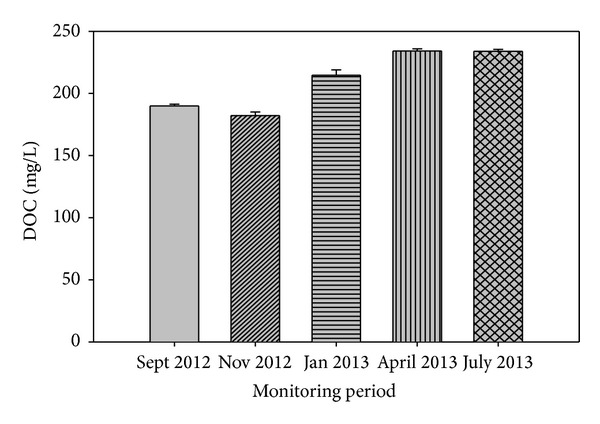
Dissolved organic carbon from peat soil cultivated with pineapple in the wet and dry seasons. (Error bars represent standard error and mean DOC are not significantly different at *P* ≥ 0.05.)

The DOC was statistically similar irrespective of the monitoring period (Figure 4). This result was expected as the peat water table was controlled to fluctuate between 50 and 60 cm in the lysimeters, so as to minimize oxygen availability for carbon decomposition in the aerobic zone. Furthermore, the restriction of soil water movement in the lysimeter may have contributed to the similarity of the DOC content. This finding is also consistent with the fact that wet and dry seasons have no significant effect on DOC production in drained peats.

The higher DOC in this study than the initial concentration (64.3 mg/L) suggests that draining peat soils accelerates their chemical oxidation. This buttresses the fact that carbon is not only lost as CO_2_ but also lost through DOC. Loss of DOC is an important indicator for carbon release, because when it is leached from the peat soils into rivers it poses environmental pollution risk. This is because DOC can react with chlorine to form trihalomethane and haloacetic acids. Although these chemicals are carcinogenic, they are not completely removed from treated water [45, 46].

## 4. Conclusion

Peat soils drained for agriculture released 218.8 t CO_2_ ha/yr under bare conditions, followed by bare peat soil treated with chloroform (205 t CO_2_ ha/yr), and peat soil cultivated with pineapple (179.6 t CO_2_ ha/yr). The lower CO_2_ emission from the peat soil cultivated with pineapple was due to the regulation by pineapple photosynthetic activity, heterotrophic respiration, and decomposition of root exudates at the rhizosphere whereas the CO_2_ emission from the bare peat fumigated with chloroform was mainly due to shrinkage and consolidation of the soil. Soil CO_2_ emission was neither affected by soil temperature nor by soil moisture but the emission seemed to be controlled by moderate soil temperature fluctuation in the wet and dry seasons. Draining peat soil affected leaching of DOC. An average 235.7 mg/L loss of DOC under bare conditions, arisen principally from microbial respiration and oxidative peat decomposition, suggests rapid decline of peat organic matter through heterotrophic microbial activities. Identification of beneficial microorganisms that reduce peat decomposition may help to minimize carbon dioxide emission from cultivated peats. Further research is needed to assess partitioning of soil CO_2_ emission at the rhizosphere, as CO_2_ emission from drained peats seems to be influenced by heterotrophic and autotrophic respiration processes.

## Figures and Tables

**Table 1:** Physical and chemical properties of a drained peat soil sampled at different depths.

Variable	Mean (0 to 10 cm)	Results per soil depth (cm)			Reported standard range
		0 to 20 cm	20 to 40 cm	40 to 60 cm	
Physical properties					
Bulk density (g/cm^3^)	0.14				0.09–0.12 [31]
Water holding capacity (%)	40.2				275–322 [31]
Moisture (%)		80.9^c^	84.9^b^	88.8^a^	90–95 [32]

Chemical properties					
pH		3.8^a^ ± 0.1	3.9^a^ ± 0.1	3.9^a^ ± 0.1	3.0–4.5 [31]
Conductivity (*μ*S/cm)		178.5^a^ ± 4.6	175.4^a^ ± 4.3	172.7^a^ ± 2.4	<200 [33]
Cation exchange capacity (cmol_(+)_/kg)		146.4^a^ ± 20.1	137.6^a^ ± 13.7	175.6^a^ ± 34.9	200 [31] 145 [33]
Total organic carbon (%)		40.0^a^ ± 0.8	39.8^a^ ± 1.4	36.5^a^ ± 1.1	12–60 [31] 20.4–38.4 [34]
Total nitrogen (%)		1.33^a^ ± 0.03	1.18^b^ ± 0.04	1.12^b^ ± 0.03	1.10–1.67 [32]
Ammonium-Nitrogen (mg/L)		138.5^a^ ± 16.2	100.0^b^ ± 4.2	94.8^b^ ± 7.7	n.a.
Nitrate-Nitrogen (mg/L)		72.0^a^ ± 5.4	48.8^b^ ± 6.3	65.8^ab^ ± 3.0	n.a.

Values (mean ± standard error) with different letters across the column are significantly different at *P* ≤ 0.05.

(Note: n.a.: not available).

**Table 2:** Day and night temperatures of the experimental site (Saratok, Malaysia).

Variable	Wet season			Dry season	
	September 2012	November 2012	January 2013	April 2013	July 2013
Mean day time temperature (°C)	26.7	29.2	29.6	26.3	27.0
Mean night time temperature (°C)	23.6	24.9	24.5	24.6	24.7
Mean day and night time temperature differences (°C)	3.1	4.3	5.1	1.7	2.3

Mean soil temperature (°C)					
Early morning	29.8^a^	30.0^bc^	28.2^bc^	30.1^a^	28.7^b^
Morning	30.8^a^	32.1^ab^	29.8^a^	30.5^a^	29.5^b^
Mid-morning to afternoon	30.9^a^	32.8^a^	30.7^a^	30.5^a^	30.6^b^
Afternoon	29.7^a^	31.1^abc^	30.5^a^	29.3^ab^	32.6^a^
Evening	29.5^a^	30.1^bc^	29.4^ab^	28.7^ab^	29.2^b^
Night	29.0^a^	29.2^c^	27.9^c^	27.7^b^	28.7^b^

Mean values with different letters within the same column are significantly different at *P* ≤ 0.05.

**Table 3:** Effect of fumigating drained peat soil with chloroform on soil microbial biomass carbon.

Monitoring cycle	Mean soil microbial biomass carbon (*μ*g C/g soil)
Initial before chloroform application	94.7^a^
September 2012	29.6^f^
November 2012	73.4^b^
January 2013	56.0^d^
April 2013	67.2^c^
July 2013	46.0^e^

Mean values with different letters are significantly different at *P* ≤ 0.05.

**Table 4:** The relationship between soil CO_2_ emission, soil temperature, and soil moisture in dry and wet seasons.

Weather season	Monitoring period	Variable	Soil temperature	Soil moisture
Wet season	September 2012		*r* = 0.2253 *P* = 0.1464	*r* = 0.1955 *P* = 0.2091
	November 2012		*r* = −0.5169 *P* = 0.0001	*r* = 0.1127 *P* = 0.4264
	January 2013	Soil CO_2_ emission	*r* = −0.4829 *P* = 0.0004	*r* = 0.2290 *P* = 0.1135
Dry season	April 2013		*r* = −0.7431 *P* = 0.0001	*r* = −0.0776 *P* = 0.5558
	July 2013		*r* = −0.5992 *P* = 0.0001	*r* = −0.2299 *P* = 0.0854
Pooling data throughout the wet and dry seasons			*r* = −0.1119 *P* = 0.0710	*r* = 0.1027 *P* = 0.0980

Note: top values represent Pearson's correlation coefficient (*r*) while bottom values represent probability level at 0.05 (*n* = 72 for each monitoring period, *n* = 360 for pooling data throughout wet and dry seasons).

**Table 5:** Soil moisture during CO_2_ measurement at different times of the day in dry and wet seasons.

Time	Wet season			Dry season	
	September 2012	November 2012	January 2013	April 2013	July 2013
	Mean soil moisture (%)				
Early morning	77.7^a^	76.7^a^	77.7^a^	77.3^a^	75.1^a^
Morning	80.4^a^	77.4^a^	78.7^a^	77.9^a^	75.5^a^
Mid-morning to afternoon	77.2^a^	78.3^a^	78.3^a^	78.0^a^	75.1^a^
Afternoon	76.2^a^	79.7^a^	77.5^a^	77.5^a^	76.7^a^
Evening	78.5^a^	77.6^a^	77.0^a^	78.4^a^	73.6^a^
Night	76.3^a^	77.9^a^	76.3^a^	78.1^a^	76.1^a^

Mean values with same letter within the same column are not significantly different at *P* ≥ 0.05.
